# Combined genetic variants of human cytomegalovirus envelope glycoproteins as congenital infection markers

**DOI:** 10.1186/s12985-015-0428-8

**Published:** 2015-11-26

**Authors:** Maria-Cristina Arcangeletti, Rosita Vasile Simone, Isabella Rodighiero, Flora De Conto, Maria-Cristina Medici, Davide Martorana, Carlo Chezzi, Adriana Calderaro

**Affiliations:** Unit of Microbiology and Virology - Department of Clinical and Experimental Medicine, University of Parma, Viale A. Gramsci, 14, 43126 Parma, Italy; Unit of Molecular Genetics, University-Hospital of Parma, Parma, Italy

**Keywords:** HCMV envelope glycoproteins, Polymorphic genes, Genetic variant combinations, Congenital infection, Predictive markers

## Abstract

**Background:**

Human cytomegalovirus (HCMV) is still considered to be the main viral cause of birth defects and long-term neurological and sensory *sequelae* following congenital infection.

Several Authors sustain a key role of HCMV envelope glycoproteins, such as gB, gN and gO - mainly involved in cell targeting, viral penetration and spread - as putative virulence factors. The genes coding for these glycoproteins possess hypervariable regions, resulting in a number of genetic variants in circulating clinical strains. Considering that the genetic polymorphisms underlying the specific differences between gB, gN and gO genotypes can influence the ability of HCMV to preferentially target specific host cells, it is very likely that they play an important role in defining HCMV infection outcome.

In the present study, we analysed HCMV gB, gN and gO gene polymorphisms in viral strains isolated from paediatric patients with congenital or post-natal infection, to investigate whether specific genetic variants may be associated with congenital infection.

**Methods:**

The restriction fragment polymorphisms of genes coding for HCMV gB (UL55), gN (UL73) and gO (UL74) were investigated by analysing viral DNA extracted from 40 urine samples of as many paediatric patients with congenital or post-natal HCMV infection. Randomly selected samples were subjected to DNA sequencing and phylogenetic analysis. Statistical analysis was performed using Fisher’s exact test to assess the significance of single and combined glycoprotein genotypes frequency distribution. Statistical significance was considered at a *P* <0.05.

**Results:**

While gB genomic variants were quite homogeneously represented in both paediatric groups, the gN4 genotype significantly prevailed in congenitally infected children (89.5 %) *vs* post-natally infected children (47.6 %), with a predominance of the gN4c variant (47.4 %). A similar trend was observed for gO3 (52.6 % *vs* 19 %).

Concerning genotypes association, a statistically significant (*P* = 0.037) gN4-gO3 combination was found specifically in the congenitally infected group.

**Conclusions:**

The results indicate that the gN4 (mostly the gN4c variant) and gO3 combined genotypes could provide useful markers of congenital infection and represent suitable candidate molecules for prophylactic vaccine preparations.

## Background

Human cytomegalovirus (HCMV), the representative member of the beta-herpesvirus, is a widespread viral agent that infects the majority of the world’s population and then establishes lifelong latency. Although infection of healthy individuals is usually asymptomatic, HCMV can be responsible for serious diseases with multi-organ involvement and frequent fatal consequences in ‘at-risk’ categories of individuals, such as those with a deficient immune system due to natural, iatrogenic (e.g. bone marrow or organ transplant patients) or acquired (e.g. HIV-infected subjects) causes [1–3]. In addition, HCMV is still considered to be the main viral cause of birth defects and long-term neurological and sensory *sequelae* following congenital infection [4–7].

The consequences of HCMV congenital disease have been reportedly considered as exceeding that caused by other childhood diseases [8] so that the virus has been assigned the highest priority for vaccine development [9] even though, to date, there is no licensed vaccine. On that basis, many studies are still addressing the characterization of HCMV strains and the mechanisms being responsible for infection in utero*,* with the goal of finding reliable markers to distinguish congenital from post-natal infections.

Many gaps remain in our knowledge about the mechanisms that determine infection outcome and the duration and severity of clinical manifestations, which may involve immunological factors of the host as well as purely viral determinants [10]. Although little data is available about the impact of HCMV virulence factors on infection outcome, several Authors sustain a key role of the HCMV envelope glycoproteins, such as gB [11–13]. Indeed, in addition to being a target of neutralising antibodies and crucial for the virus interaction with cell receptors, gB is encoded by the UL55 gene presenting a number of polymorphic regions which account for its genotypic and phenotypic variability, giving rise to four principal subtypes (gB1-gB4) of HCMV circulating strains [14, 15].

More recently, other envelope glycoproteins have been indicated as putative HCMV virulence factors, such as the glycoproteins N (gN) and O (gO) [16–18]. Similarly to gB, the genes (UL73 and UL74) coding for these glycoproteins possess hypervariable regions, resulting in a number of gN and gO subtypes. The gN variants are as follows: gN1, gN2, gN3a, gN3b, gN4a, gN4b, gN4c; in relation to gO, four main clades have been described, gO1-gO4, which can be further divided into seven genetic variants (gO1a, gO1b, gO1c, gO2a, gO2b, gO3, gO4) [19]. Glycoprotein N is involved in virus attachment to the host cell and viral spread, while gO participates in the fusion of the viral envelope to the host cell membrane, promoting HCMV penetration, envelope acquisition and release [16, 17, 20–22].

Considering that the genetic polymorphisms underlying the specific differences between gB, gN and gO subtypes can influence the ability of HCMV to preferentially target specific host cells, it is very likely that they play an important role in defining HCMV infection outcome [12, 16, 23]. It is also of note that genes encoding the above-mentioned glycoproteins generally act in a coordinated and synergistic way [17, 19, 24]. Thus, in the quest to identify predictive biomarkers of infection outcome, studies addressing the combined polymorphic patterns of HCMV genes encoding envelope glycoproteins are much more representative than those focussed on single polymorphisms.

Based on the aforementioned notions, the present study focussed on HCMV gB, gN and gO gene polymorphisms in viral strains present in urine samples of paediatric patients with congenital or post-natal HCMV infection, to investigate whether the prevalence of combined genetic variants may be associated with congenital infection.

## Results

### Restriction Fragment Length Polymorphism (RFLP) patterns of polymorphic HCMV genes encoding gB, gN and gO glycoproteins in the studied population

**Fig. 1:**
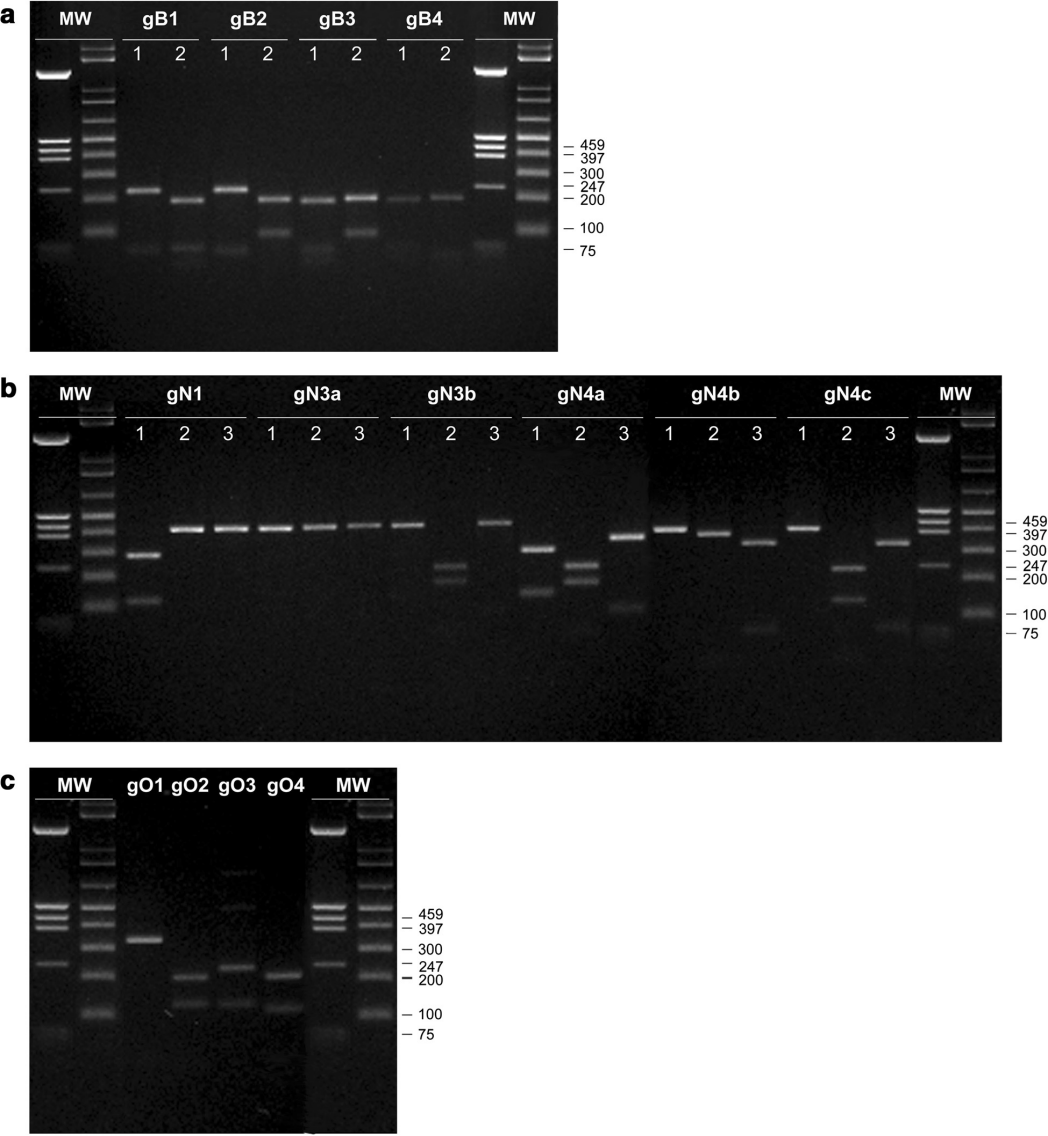
Representative RFLP patterns of HCMV gB, gN and gO glycoproteins in the studied population. RFLP analysis was performed on the PCR-amplified gB (**a**), gN (**b**) and gO (**c**) sequences obtained from the patient-derived HCMV strains. Each sample was digested with restriction enzyme combinations as detailed in Tables 1, 2 and 3. In the upper side of panels **a** (gB) and **b** (gN), the numbers “1, 2” (panel **a**), and “1, 2, 3” (panel **b**) indicate the restriction enzymes used (gB: 1 = *Rsa*I; 2 = *Hinf*I; gN: 1 = *Sac*I; 2 = *Sca*I; 3 = *Sal*I). A single digestion with *Hpa*II was used to characterise the gO gene products (panel **c**). The genotypes corresponding to each digest are displayed at the top of the lanes. Lanes *MW* molecular weight markers

Genetic polymorphisms of HCMV envelope glycoproteins B, N and O in the paediatric cohort considered in this study were analysed by RFLP. The patterns of the expected fragments obtained upon endonuclease digestion (Tables 1, 2 and 3) are shown in Fig. 1 for gB (panel a), gN (panel b) and gO (panel c) genotypes. As observed, all the expected genotypes are present for gB and gO; concerning gN, only the gN2 genotype was absent in the studied population.

**Table 1:** Amplicon sizes and restriction endonuclease patterns of gB genomic variants

gB genotype and PCR product size (bp)	(*) Fragment sizes (bp)	
	*Rsa*	*Hinf*I
1 (320)	239, 66	202, 67
2 (320)	239, 63	202, 100
3 (320)	195, 62	202, 97
4 (320)	195, 66	202, 67

(*) For each of the glycoprotein gene amplification products, the restriction pattern was obtained upon digestion with the endonuclease combinations indicated in the related panels

**Table 2:** Amplicon sizes and restriction endonuclease patterns of gN genomic variants

gN genotype and PCR product size (bp)	(*) Fragment sizes (bp)		
	*Sac*I	*Sca*I	*Sal*I
1 (420)	297, 123	420	420
2 (417)	229, 123, 65	417	296, 121
3a (420)	420	420	420
3b (420)	420	221, 172, 27	420
4a (414)	291, 123	221, 166, 27	341, 73
4b (414)	414	387, 27	341, 73
4c (414)	411	239, 172	338, 73

Pignatelli et al. [22]

(*) For each of the glycoprotein gene amplification products, the restriction pattern was obtained upon digestion with the endonuclease combinations indicated in the related panels

**Table 3:** Amplicon sizes and restriction endonuclease patterns of gO genomic variants

gO genotype and PCR product size (bp)	(*) Fragment sizes (bp)
	*Hpa*II
1 (372)	372
2 (372)	203, 141
3 (372)	229, 141
4 (372)	203, 115

(*) For each of the glycoprotein gene amplification products, the restriction pattern was obtained upon digestion with the endonuclease indicated in the related panel

### HCMV DNA sequencing and phylogenetic analysis

**Fig. 2:**
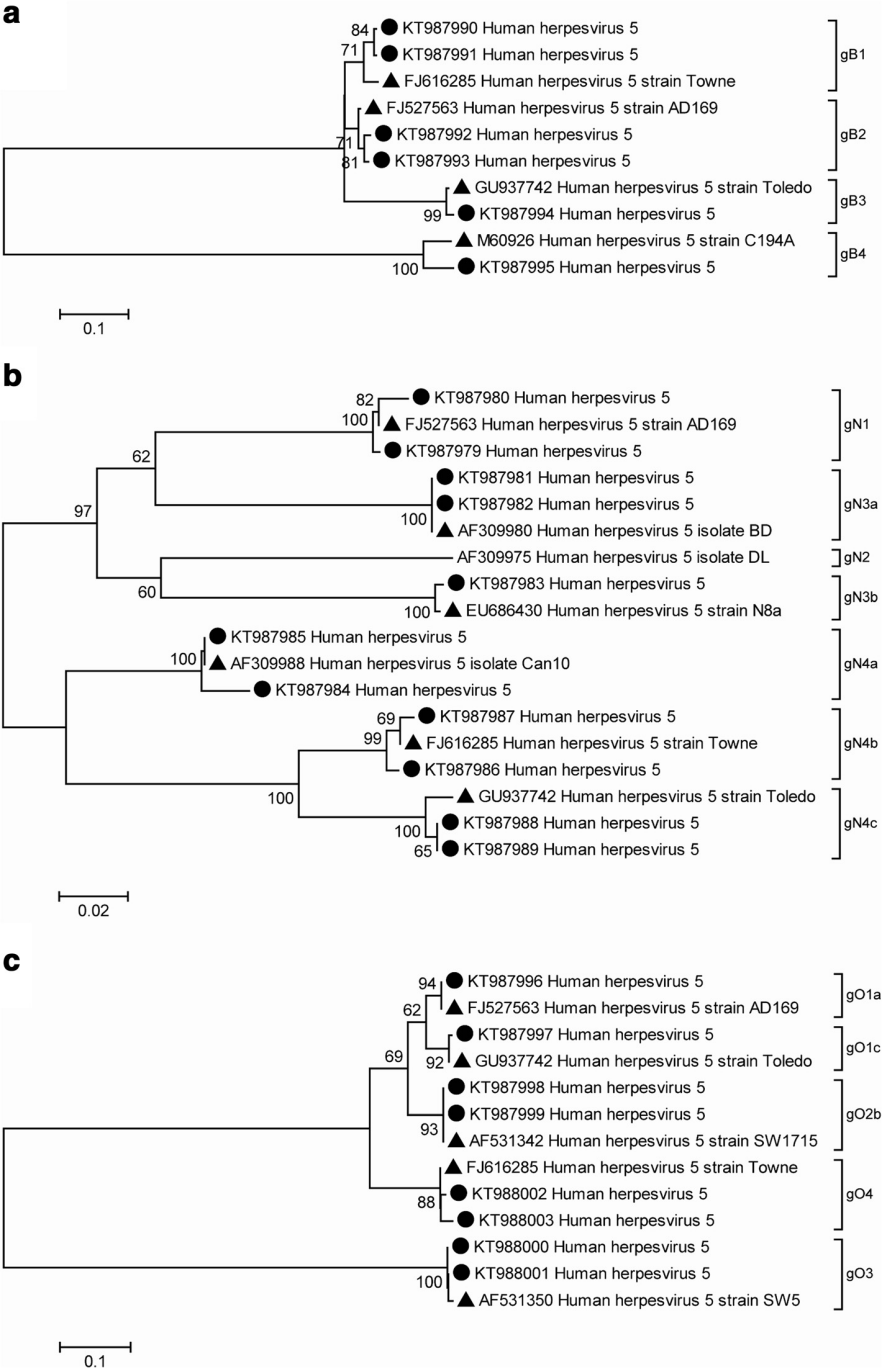
Phylogenetic analysis of HCMV gB, gN and gO sequences from congenitally and post-natally infected children. Phylogenetic analysis was based on partial UL55 (496 nucleotides) (panel **a**), UL73 (313 nucleotides) (panel **b**) and UL74 (275 nucleotides) (panel **c**). Bootstrap values >60 % are indicated. Scale bars indicate the number of nucleotide substitutions per site. HCMV gB, gN and gO genotypes are indicated on the right side of the dendrograms. Italian strain sequences and reference sequences are indicated with a circle and a triangle, respectively

DNA sequencing and phylogenetic analysis of randomly selected HCMV gB, gN and gO genotypes from the studied population were performed to confirm the RFLP assignments. The phylogenetic analysis of the nucleotide sequences of the three glycoproteins (Fig. 2) was in agreement with the results obtained by the RFLP method. HCMV gB, gN and gO genotypes identified by RFLP segregated highly correlated with the corresponding reference strains (Fig. 2a: gB; b: gN; c: gO), with a nucleotide identity range from 97 to 99 % for gB, 99 to 100 % for gN, 99 to 100 % for gO.

Furthermore, while endonucleases are only available for the four main clades of the gO genotype (gO1 to gO4), phylogenetic clustering allowed us to detect three additional genetic variants (gO1a, gO1c and gO2b).

### Analysis of HCMV gB, gN, gO single genotypes distribution in the studied population

**Fig. 3:**
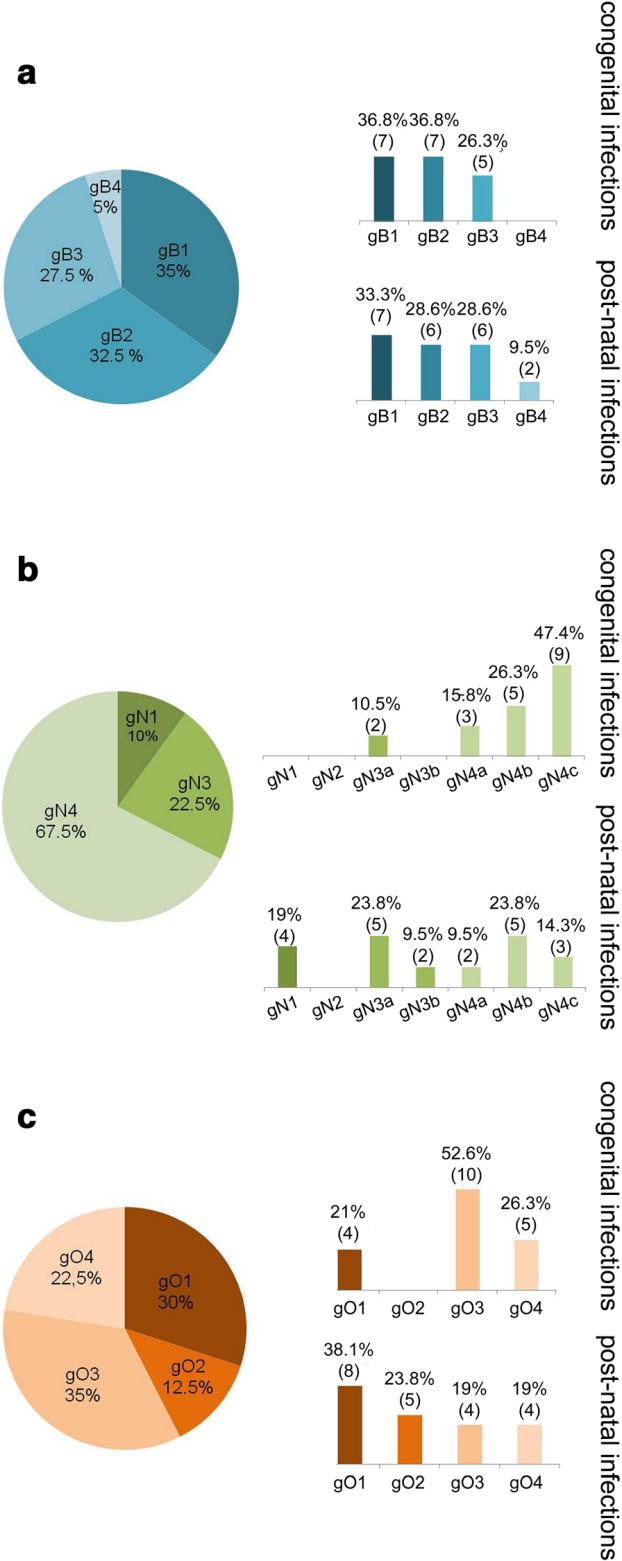
Glycoprotein B, N and O genotype distributions in congenitally and post-natally infected children. The overall distributions of gB, gN and gO genotypes are shown in the pie charts of panels **a**, **b** and **c**, respectively; the subdivision of gB, gN and gO variants among congenitally and post-natally infected children is displayed in the adjacent bar charts. The number of subjects infected with the specific genotypes and the related percentages are indicated at the top of each bar

The overall distribution of the considered glycoprotein genotypes and their specific partitioning between the congenitally and post-natally infected groups are shown in Fig. 3. With regard to gB (Fig. 3a), our data confirm the presence of all gB genotypes in the whole paediatric population (pie chart). The genomic variants gB1, gB2 and gB3 were quite homogeneously represented (35, 32.5 and 27.5 %, respectively), while gB4 was poorly present (5 %). A similar trend was maintained in both groups (congenitally and post-natally infected patients), except for the gB4 genotype which was absent in the congenitally infected group.

Concerning gN (Fig. 3b), a clear prevalence of the gN4 genotype (67.5 %) was found in the whole paediatric population (pie chart). Interestingly, the analysis of the gN distribution between the two paediatric groups shows a significant predominance of the gN4 genotype in the congenitally infected children (89.5 %, not shown as a whole), with a prevalence of the gN4c variant (47.4 %), compared with post-natally infected children (gN4: 47.6 %, not shown as a whole; gN4c: 14.3 %). The gN1, gN2 and gN3b genotypes were not observed in the first group, while only gN2 was absent in the second group.

Finally, concerning gO (Fig. 3c), the overall distribution revealed a dominance of the gO1 (30 %) and gO3 (35 %) genotypes over gO2 and gO4 (12.5 and 22.5 %, respectively) (pie chart). However, the trends were distinct for the congenitally and post-natally infected paediatric groups: a significant predominance of gO3 (52.6 %) was only observed in the congenitally infected newborns, compared with just 19 % in the post-natally infected children. A slight prevalence of the gO1 genotype (38.1 %) was present in the latter group.

Statistical analysis of the frequency distribution of HCMV gB, gN and gO genotypes in the congenitally *vs* post-natally infected children showed a significant association of gN4 (*P* = 0.006), mostly the gN4c variant (*P* = 0.037), with congenital infections; a similar trend was found for the gO3 genotype (*P* = 0.045).

### Combined polymorphic patterns of genes encoding HCMV gN and gO glycoproteins in congenitally and post-natally infected children

**Fig. 4:**
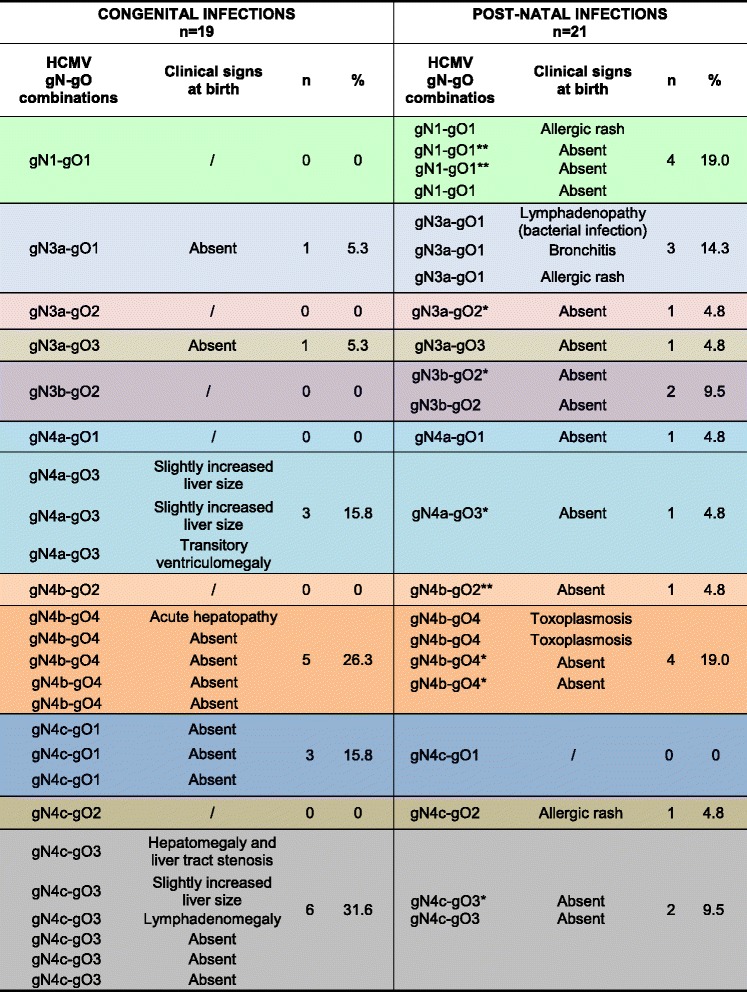
Combined patterns of HCMV gN and gO genotypes and relationships with clinical pictures at birth. * Premature birth. ** Small size at birth

This study also looked at the combinations of glycoprotein genotypes present in the considered population (Fig. 4). Only the combinations of gN and gO glycoprotein gene polymorphisms were investigated, since these showed the most relevant and distinct patterns between the two paediatric groups when analysed singularly. The most significant difference between the groups was found in the prevalence of the gN4-gO3 combination in the congenitally infected group, constituting 47.4 % of cases compared to 14.3 % in the post-natally infected children. The observed differences in the occurrence of the gN4-gO3 combined genotypic pattern in congenital *vs* post-natal infections were statistically significant (*P* = 0.037).

In the post-natally infected group, the most frequently observed HCMV mixed genotypes were gN1-gO1 and gN4b-gO4 (being equally prevalent, at 19 % each).

## Discussion

HCMV congenital infection is one of the major causes of birth defects and late neurological and sensory *sequelae* in children. Around 0.2–2 % of newborns acquire HCMV infection in utero [25, 26], but only 10–15 % of children with congenital infection show relevant clinical signs at birth [5, 27–29].

HCMV envelope glycoproteins showing genetic polymorphisms among circulating strains have been considered as potential virulence markers, and may be responsible for differential HCMV tropism to specific cell types and differential ability to disseminate and interfere with normal tissue development [13, 16, 21, 23]. A number of studies have focussed on these viral components, although most of them have only addressed singular glycoprotein subtypes. In particular, UL55 (coding for gB), the less polymorphic gene among those coding the main envelope glycoproteins, has been analysed in different categories of subjects at risk of developing diseases upon HCMV infection [30–32]. Some Authors found that all the four dominant gB genotypes can be transmitted to the *foetus* in the case of congenital infections [33–35], while others found gB1 to be the prevalent genotype in congenital infections [11, 36]; a prevalence of certain genotypes in patient cohorts characterised by distinct geographical or ethnic contexts has been also reported [37, 38]. Collectively, these data do not favour the hypothesis that specific gB genotypes alone could provide reliable markers of congenital infection, partially due to the low genetic variability of the *locus* (9.5 %) and also because most of the gB subtypes demonstrate a similar probability of being associated with congenital infection [33]. Conversely, the UL73 gene, coding for the envelope glycoprotein N, possesses highly hypervariable regions (50 % variability). The UL74 gene coding for gO also shows a considerable (30 %) variability, but has not been largely analysed in this category of at-risk subjects [38]. In summary, although no conclusive results concerning the infection outcome and tissue tropism of HCMV variants carrying specific glycoprotein polymorphisms have been produced to date, the genetic characterisation of the infecting HCMV strains appears useful for the infection prognosis and, thus, remains the focus of many current studies [13]. As already mentioned, it is unlikely that just a single polymorphic gene product is responsible for a specific infection outcome and/or tissue tropism, especially in the case of a viral agent as complex as HCMV. Instead, it is probable that the viral phenotype is determined by a combination of polymorphic genetic *loci* that operate synergistically [19, 24]; this is likely the case for UL73 and UL74 (coding for gN and gO, respectively).

In this study we have addressed the polymorphisms of HCMV genes encoding gB, gN and gO envelope glycoproteins in a cohort of paediatric patients with congenital or post-natal HCMV infection, in order to assess whether the presence of specific genetic combinations of viral glycoprotein subtypes may constitute reliable markers of infection outcome. By first analysing the distribution of single genotypes, we found a significant predominance of gN4 in congenital infections, in accordance with other studies [17, 18], implicating this genotype as a potential prognostic marker of HCMV congenital infection. Furthermore, our results support a significant association of congenital infections with the gN4c genetic variant; a similar trend was found for gO3. The RFLP results were further confirmed by applying DNA sequencing and phylogenetic analysis to randomly selected samples.

With regard to combined genotypes, we detected a statistically significant association of gN4 and gO3 genetic variants in congenitally infected children; it is noteworthy that, among those presenting clinical signs at birth (7/19), all were gN4 and nearly all (6 out of 7) showed the gN4-gO3 combination. On the other hand, most of the post-natally infected infants did not show any clinical signs at birth; a minority of them presented clinical signs not compatible with HCMV infection (see Fig. 4).

It is also of interest to consider that although congenitally infected newborns with clinical signs are at higher risk of developing long-term neurological and sensory *sequelae* (about 49 % on average) [6, 39–41], a considerable portion of asymptomatic babies (about 13.5 %) eventually manifest such problems [6, 26]. Moreover, the above mentioned percentages may be under-estimated in the literature because data on late disabilities are often incomplete and follow-up periods too short to identify late *sequelae.*

Thus, the development of prognostic markers of HCMV congenital infection is of great importance for the identification and characterisation of predominant genetic variants of HCMV in at-risk patients, as well as for the development of protective vaccines. With regard to the latter, the major problem concerns the high variability of circulating strains that are differentially involved in clinical manifestations with different degrees of severity, making it highly difficult to choose a viral product that would present the highest level of safety whilst effectively eliciting humoral as well as cellular immunity. In this context, it is noteworthy that the gN viral glycoprotein is one of the viral products eliciting the highest neutralising antibody response and that anti-gN4 immunity also seems to protect against frequent re-infection [42]; it is also likely that specific genetic variants of the gN4 protein (such as gN4c as shown here) could enhance this relevant feature even further.

Another interesting aspect of HCMV congenital infections is the possible presence of multiple viral strains. Indeed, although a number of studies have outlined the notion that mixed infections are more frequently found in adult immunocompromised hosts [15, 43], more recently published studies have also described the presence of multiple HCMV strains in congenital infections at a relatively high rate (15–46 %) [44, 45].

Surprisingly, we didn’t find mixed HCMV infections in our study population. This might be attributed to the fact that our sample size, although sufficient to obtain statistically significant results, was quite small, thus associated with a lower probability of detecting mixed infections; for instance, similar results were also obtained recently by other Authors [7]. Moreover, it has been reported that a mixed infection has a higher chance of being detected if the less represented genotype constituted at least 25 % of the total viral population [7]; thus, we can speculate that additional genotypes may have been present in a smaller proportion in our study population. Another interesting notion, highlighted by Ross and collaborators [44], is that in some cases only one type of HCMV genotype could be found in a specific biological sample, while other genotypes could prevail in samples obtained from different compartments of the same child. This observation could also account for the apparent absence of mixed genotypes in our study population as only urine samples were tested.

Work is in progress in our laboratory to collect and analyse more than one type of biological sample from HCMV infected babies.

In summary, identification of the predominant molecular combinations of glycoprotein subtypes in congenital HCMV infections, like gN4-gO3 as shown in the present study, is crucial for unravelling the complicated interplay between glycoprotein genetic variation and HCMV pathogenicity. Identification of such combinations will also permit a more accurate prenatal diagnosis and focussed, long-term follow-up care for infected babies.

## Conclusions

This study is one of few to address not only the polymorphisms of individual genes encoding HCMV glycoproteins, but also their combination in one of the most important categories of at-risk subjects, i.e. congenitally infected children.

Although the sample size considered in the present study is limited, it has sufficient power to detect significant differences in the two paediatric groups considered. Indeed, we reveal that the gN4-gO3 genotype association is statistically significant in the congenitally infected cohort.

These results indicate that the gN4 (mostly the gN4c variant) and gO3 glycoprotein genotypes could provide reliable markers of congenital infection. The identified variants could also constitute ideal candidates for prophylactic measures that aim to counteract the onset of late disabilities in congenitally infected children.

## Materials and methods

### Patients and clinical samples

Forty children with congenital (*n* = 19) or post-natal (*n* = 21) HCMV infection hospitalised at the University-Hospital of Parma, Italy, over a 15-year period were included in this retrospective study (formal consent was not required). Inclusion into either the congenitally or post-natally infected group was based on the positive or negative outcome of a HCMV urine test performed at birth, according to the rules indicated by the Centers for Disease Control and Prevention (Atlanta, USA; see: http://www.cdc.gov/cmv/testing-diagnosis.html). Detailed characterizations of both the study populations are listed below.

#### Congenitally infected children

Age range at the time of HCMV urine positive testing: 1–5 days after birth.

Sex: 9 females; 10 males.

Mothers’ serostatus during pregnancy: HCMV seroconversion during pregnancy: 16; no data available: 3.

Reasons for the request of HCMV urine testing at the time of birth: 16 infants born from HCMV-seroconverted mothers during pregnancy (one neonate presented cervical lymphadenomegaly; three presented a mild liver enlargement; 12 had normal parameters at birth). Of the three cases with no data available on the mothers’ serostatus: one newborn presented acute hepatopathy; one presented transitory ventriculomegaly; one presented hepatomegaly, liver tract stenosis and increased transaminase levels.

#### Post-natally infected children

Age range at the time of HCMV urine negative testing: 3–9 days after birth.

Age range at the time of HCMV urine positive testing: 2 months to 5 years after birth (most likely primary perinatal infections via breast milk or environmental primary infections).

Sex: 12 females; 9 males.

Mothers’ serostatus: presence of anti-HCMV IgG antibodies before pregnancy: 10; anti-HCMV antibodies negative during pregnancy: 2; no data available: 9.

Reasons for HCMV urine testing at the time of birth: in two cases it was requested to make a differential diagnosis with *Toxoplasma gondii* infection that occurred during pregnancy; three newborns presented a skin rash finally attributed to an allergic reaction; five newborns presented increased transaminase levels, but exhibited normal liver and spleen dimensions; six newborns had a premature birth; three newborns presented a small size at birth, one newborn presented lymphadenopathy, later attributed to *Streptococcus pyogenes* infection; one newborn presented a respiratory syndrome, later diagnosed as a *Staphylococcus aureus* infection.

None of the newborns considered in this study presented neurological signs or sensory impairment at birth. Medical follow-up of the infected paediatric patients during early childhood was not available.

Forty HCMV-positive urine samples (obtained from each of the aforementioned paediatric subjects) were considered in the study. Laboratory tests were performed at the Virology Unit of the University-Hospital of Parma; samples underwent a rapid culture test using human fibroblasts, and HCMV immediate-early antigens were detected in infected cells using an indirect immunofluorescence assay. After initial testing, urine sample were then stored at −80 °C until later use.

The samples were subjected to DNA extraction followed by gB, gN and gO genotyping.

### DNA extraction and amplification

A 1 ml aliquot of each urine sample was used for total DNA extraction employing the commercial kit “QIAamp Blood Mini kit” (Qiagen), according to the manufacturer’s instructions. The extracted DNA was subjected to polymerase chain reaction (PCR) using primers for gB, gN and gO specific regions and amplification conditions previously described [12, 16, 22]. The laboratory strains AD169 (ATCC VR-538) and Towne (ATCC VR-977) were used as positive controls.

### Restriction fragment length polymorphism

The considered HCMV glycoprotein gene fragments were digested with the following combinations of restriction enzymes: *Rsa*I (*Rhodopseudomonas sphaeroides*) and *Hinf*I (*Haemophilus influenzae*) [gB]; *Sac*I (*Streptomyces achromoqenes*), *Sca*I (*Streptomyces caespitosus*) and *Sal*I (*Streptomyces albus* G.) [gN]; *Hpa*II (*Haemophilus parainfluenzae*) [gO]. Restriction enzymes were obtained from Thermo Scientific. Digestions were performed according to manufacturer’s instructions. The patterns of restriction fragments were analysed by a 2 % agarose gel electrophoresis. Molecular weight markers were from Invitrogen (1 Kb DNA ladder; 100–12,000 bp) and from Nanogen Advanced Diagnostics (Marker Hinf I; 46–1380 bp).

### DNA sequencing and phylogenetic analysis

DNA sequencing and phylogenetic analysis were performed using a random selection of 6 gB, 11gN and 8 gO HCMV clinical strains previously characterized by RFLP. PCR products were purified (Qiaquick Gel Extraction Kit, Qiagen) and sequenced with an automated sequencer (CEQ 2000XL DNA Analysis System – Beckman Coulter). For DNA sequencing, the same primers used to amplify gB, gN and gO genetic *loci* (UL55, UL73, UL74) were employed. The electropherograms were analysed and edited using the DNA Sequencing Analysis software Version 3 (Applied Biosystem). The genetic identity of each strain was determined by comparison with reference strains available in GenBank database (US National Center for Biotechnology Information, NCBI). The partial gB, gN and gO genotype sequences were deposited in GenBank under the accession numbers listed hereafter: KT987990, KT987991, KT987992, KT987993, KT987994, KT987995 for UL55; KT987979, KT987980, KT987981, KT987982, KT987983, KT987984, KT987985, KT987986, KT987987, KT987988, KT987989 for UL73; and KT987996, KT987997, KT987998, KT987999, KT988000, KT988001, KT988002, KT988003 for UL74. Multiple sequence alignments and phylogenetic tree constructions were performed with MEGA v6.0 [46], applying the maximum-likelihood method. Tree reliability was assessed by bootstrap re-sampling over 1000 replicates. Reference strains that shared >97 % nucleotide identity with our sequences were retrieved from the GenBank database.

### Statistical analysis

Data were analysed using the SPSS (Statistical Package for Social Science) software (Version 20.SPSS). Statistical analysis was performed using Fisher’s exact test to assess the significance of single and combined HCMV glycoprotein genotypes frequency distribution in congenitally *vs* post-natally infected groups. Statistical significance was considered at a *P* <0.05.

## Abbreviations

g: glycoprotein
HCMV: human cytomegalovirus
PCR: polymerase chain reaction
RFLP: restriction fragment length polymorphism
